# A highly conserved arginine residue of the chitosanase from *Streptomyces* sp. N174 is involved both in catalysis and substrate binding

**DOI:** 10.1186/1471-2091-14-23

**Published:** 2013-09-16

**Authors:** Marie-Ève Lacombe-Harvey, Mélanie Fortin, Takayuki Ohnuma, Tamo Fukamizo, Thomas Letzel, Ryszard Brzezinski

**Affiliations:** 1 Département de Biologie, Centre d’Étude et de Valorisation de la Diversité Microbienne, Faculté des Sciences, Université de Sherbrooke, Sherbrooke, QC, Canada; 2 Department of Advanced Bioscience, Kinki University, Nara, Japan; 3 Chair of Urban Water Systems Engineering, Technische Universität München, Garching, Germany

**Keywords:** Chitosanase, Glycoside hydrolase family GH46, Substrate inhibition, Inverting mechanism, Enzyme-substrate interaction, Arginine

## Abstract

**Background:**

*Streptomyces* sp. N174 chitosanase (CsnN174), a member of glycoside hydrolases family 46, is one of the most extensively studied chitosanases. Previous studies allowed identifying several key residues of this inverting enzyme, such as the two catalytic carboxylic amino acids as well as residues that are involved in substrate binding. In spite of the progress in understanding the catalytic mechanism of this chitosanase, the function of some residues highly conserved throughout GH46 family has not been fully elucidated. This study focuses on one of such residues, the arginine 42.

**Results:**

Mutation of Arg42 into any other amino acid resulted in a drastic loss of enzyme activity. Detailed investigations of R42E and R42K chitosanases revealed that the mutant enzymes are not only impaired in their catalytic activity but also in their mode of interaction with the substrate. Mutated enzymes were more sensitive to substrate inhibition and were altered in their pattern of activity against chitosans of various degrees of deacetylation. Our data show that Arg42 plays a dual role in CsnN174 activity.

**Conclusions:**

Arginine 42 is essential to maintain the enzymatic function of chitosanase CsnN174. We suggest that this arginine is influencing the catalytic nucleophile residue and also the substrate binding mode of the enzyme by optimizing the electrostatic interaction between the negatively charged carboxylic residues of the substrate binding cleft and the amino groups of GlcN residues in chitosan.

## Background

In the past decade, several studies aimed to improve our understanding of the mechanisms of enzymatic hydrolysis of chitosan, a polycationic polysaccharide containing β-1,4 linked residues of d-glucosamine (GlcN) with a minor proportion of *N*-acetyl-d-glucosamine (GlcNAc) [1]. The chitosanase originating from *Streptomyces* sp. N174 (CsnN174), which belongs to the glycoside hydrolases family 46 (GH46), is among the best characterized [2-4]. This enzyme is an *endo*-type hydrolase and proceeds *via* an inverting mechanism in which Glu22 acts as the general acid and Asp40 as the general base/nucleophile [4,5]. From a structural view, the CsnN174 consists of two globular domains mainly constituted of α-helices. The connection of the two domains by a helix backbone generates the substrate binding cleft [6]. The structural features of CsnN174 are not only shared by GH46 members, but also by GH22, GH23 and GH24 lysozymes, as well as GH19 chitinases, all members of the “lysozyme superfamily” [2,6-9]. This list could be extended toward GH80 family based on primary sequence similarities [10].

It is now generally recognized that some residues of the catalytic cleft of glycoside hydrolases, other than the catalytic residues, might play an essential role in enzyme action. Such residues can be involved in enzyme-substrate interaction, in structure stabilisation, but also in the creation of interaction networks essential for catalysis [6,11-13]. In CsnN174, examination of the microenvironment of the proton donor Glu22 revealed that this glutamate does not work independently but requires assistance from an interaction network involving three other residues (Arg205, Asp145 and Arg190) to achieve efficient catalysis [13]. Similar networks have also been observed in other members of the lysozyme superfamily, *Bacillus circulans* MH-K1 chitosanase (GH46), T4 lysozyme (GH24) and barley chitinase (GH19) [13]. In order to verify if a similar kind of interaction is needed to sustain the catalytic potential of the general base residue, we examined the microenvironment of Asp40 of CsnN174 and found that an arginine (Arg42) is highly conserved among the GH46 family of chitosanases. Implication in the catalytic function of an arginine which lies in an analogous position has been reported in the *Bacillus circulans* MH-K1 chitosanase [14]. The present work clarifies the contribution of Arg42 to the enzymatic activity of the *Streptomyces* sp. N174 chitosanase.

## Methods

### Materials and reagents

The chitosan substrate (84%-*N*-deacetylated) used for routine enzyme assays was from Sigma-Aldrich (St. Louis, MO). Restriction enzymes were from New England Biolabs (Beverly, MA). All reagents and enzyme substrates were of analytical grade. Culture media components were from Difco (Mississauga, Ontario, Canada).

### Bacterial strains and plasmids

*E. coli* strain DH5α (F- φ80*lac*ZΔM15 Δ(*lac*ZYA-*arg*F)U169 *rec*A1, *end*A1, *hsd*R17(rk^-^, mk^+^) *pho*A, *sup*E44, *thi*-1, *gyr*A96, *rel*A1 λ^-^) was used for plasmid propagation. *E. coli* strain XL10-Gold Ultracompetents Cells (Tet^r^ Δ(*mcrA*)*183* Δ(*mcrCB-hsdSMR-mrr*)*173 endA1 supE44 thi-1 recA1 gyrA96 relA1 lac* Hte [F’ *proAB lacI*^q^*Z*Δ*M15* Tn*10* (Tet^r^) Amy Cam^r^]) (Stratagene, CA, USA) were used for plasmid propagation and as hosts for induction and isolation of recombinant chitosanases from *E. coli.* Recombinant strains of *Streptomyces lividans* TK24 were used for chitosanase production [5]. The vector pUC19 (for induction and isolation of recombinant chitosanase from *E. coli* cells and for saturation mutagenesis of Arg42 residue) was described previously [15]. The shuttle vector pFDES, a derivative of pFD666, was used as vector for expression of mutated chitosanase genes [16-18].

### Saturation mutagenesis of R42 residue

In order to facilitate the saturation mutagenesis procedure, derivatives of the previously described pUC19-csnN174 and pUC19-csnN174-D40G plasmids have been generated [17]. These derivatives, named pUC19-csnN174-AN and pUC19-csnN174-D40G-AN, harboured two unique restriction sites *Nco*I and *Age*I*,* respectively upstream and downstream from the Arg42 codon. These sites have been created by silent mutagenesis, using the method involving polymerase chain reaction (PCR) performed with the *Easy-A® High-Fidelity PCR Cloning Enzyme* (Stratagene, CA, USA) [19]. The mutated DNA sequences were confirmed by DNA sequencing.

Site-specific saturation mutagenesis of the CsnN174 Arg42 codon was achieved by GENEART Inc. (Regensburg, Germany) using the pUC19-CsnN174-AN or pUC19-Csn N174-D40G-AN constructs as templates. Variants of Arg42 codon were created by introducing the synthetic *Nco*I/*Age*I 128 bp fragments of the *csnN174* gene (wild type or D40G mutant) into pUC19-CsnN174-AN or pUC19-CsnN174 D40G-AN using *Nco*I and *Age*I restriction sites. The final constructs were verified by sequencing at GENEART Inc (Regensburg, Germany). All Arg42 mutants were transformed into *E. coli* strain XL10-Gold Ultracompetents Cells. The *csnN174* R42E, R42K, D40G+R42E and D40G+R42K mutated genes were expressed in *S. lividans* TK24. Each of these genes was excised from corresponding pUC19-CsnN174-AN or pUC19-CsnN174 D40G-AN construct by *Sma*I and *Hin*dIII digestion. The resulting 1142 bp fragments encoding the mutated chitosanases were subcloned into the pFDES vector previously digested with *Sca*I and *Hin*dIII, and transformed into *S. lividans* TK24 for expression.

### Induction and isolation of recombinant chitosanases from *E. coli* cells

Starter cultures of *E. coli* strain XL10-Gold cells carrying the pUC19, pUC19-Csn WT or pUC19-CsnN174 R42X-AN vectors were prepared by inoculating 10 ml of sterile Luria broth supplemented with 100 μg/ml ampicillin and incubated overnight with shaking (250 rpm) at 37°C. Then, cultures of 100 ml were inoculated with 5 ml of starter culture and incubated with shaking (250 rpm) at 37°C until optical density reached 0.6 at 600 nm. IPTG was added to a final concentration of 1 mM and the cultures were further incubated for 3.5 hours at 37°C. The cells were harvested by centrifugation at 4000 rpm at 4°C for 10 minutes. Cell pellets were washed twice with 50 mM sodium acetate buffer (pH 5.5) and suspended in 500 μl of buffer supplemented with *Complete® Protease Inhibitor Cocktail* (Roche, Mannheim, Germany) and transferred to *FastPrep®* impact-resistant 2.0 ml tubes (Qbiogene, Carlsbad, CA) containing 250 mg of 0.1 mm glass beads. Cell disruption was carried out in ice bath using *Fast Prep®* FP120 Cell Disrupter (QBiogene, Carlsbad, CA) for 40 sec at 6.5 m/sec speed. After centrifugation (13 000 rpm for 10 min), the supernatant was assayed for protein concentration and chitosanase activity. Protein concentration was determined according to Bradford [20] using bovine serum albumin as standard. All the induction experiments were done in triplicate.

### Chitosanase assay in proteins extracts from *E. coli* cells

Chitosanase activity in protein extracts was determined as described [21] except that the reaction time was of 120 min. Comparisons of chitosanase activity among the wild-type and Arg42-mutated enzymes were made with ANOVA test (*P*<0.05) followed by a Dunnett’s post test.

### Chitosanase purification and assay

Wild-type, D40G, R42E, R42K, D40G+R42E and D40G+R42K chitosanases were purified from recombinant *S. lividans* TK24 culture supernatants as previously described [17]. Chitosanase and protein assays were as described [21]. Specific activities were determined at a single chitosan concentration (800 μg/ml).

Kinetic parameters were determined using chitosan as substrate at 37°C in 50 mM sodium acetate buffer (pH 5.5). 0.4 ml reaction mixtures were set up containing 8 to 16 different concentrations (from 0.02 to 0.8 mg/ml) of chitosan in 8 replicas using micro-titer plates. Protein concentration and reaction time were adjusted to obtain similar overall hydrolysis levels for all studied proteins. Reaction time was of 10 min for wild-type or of 20 min for D40G, R42E and R42K. Release of reducing sugars was monitored as described previously [21]. *K*_m_, *k*_cat_ and *K*_*i*_ values were calculated by nonlinear regression, fitting the experimental data to the enzyme kinetic-substrate inhibition equation in Prism Software (GraphPad Prism, version 5.0 for Windows, San Diego, CA, USA) [22]:

$$Y=V_{\max}*X/\left[K_{m}+X*\left(1+X/K_{i}\right)\right]$$

The effect of the degree of *N*-deacetylation of chitosan on wild-type, R42E and R42K chitosanases specific activities was studied using chitosans with varying degrees of *N*-deacetylation as substrates at a constant concentration of 800 μg/ml in 6 replicas. Reaction time was of 10 min for wild-type or of 20 min for R42E and R42K. Release of reducing sugars was monitored as described previously [21]. Chitosans of degree of *N*-deacetylation of 97%, 94% and 86% were obtained respectively from Shanghai Freeman Americas (Edison, NJ, USA), Marinard Biotech (Rivière-au-Renard, Quebec, Canada) and ISM Biopolymer (Granby, Quebec, Canada). Chitosan of degrees of *N*-deacetylation of 86%, 72%, 65% and 62% were obtained by treating chitosan from Sigma-Aldrich (84%-*N*-deacetylated) with acetic anhydride, as described [23]. The degree of *N*-deacetylation of all chitosan samples was determined by ^1^H-NMR [24].

### Enzymatic reaction samples for the real-time mass spectrometric assay

#### Hydrolysis reaction with wild-type and mutant CsnN174

Samples were prepared in duplicate using 10 mM ammonium acetate (pH 5.2) aqueous solutions containing 5 nM wild-type CsnN174, 62.5 nM R42E mutant, 62.5 nM R42K mutant, respectively, and 25.0 μM (GlcN)_6_. Each experiment was immediately started by adding substrate to the enzyme solution. The data correction with ionization factors was performed as described previously [25,26].

Concentrations of glucosamine dimer, trimer, tetramer, hexamer were determined from intensity counts of time-course graphs of (GlcN)_6_ hydrolysis monitored by real-time mass spectrometry. Oligomers concentration data obtained were corrected in order to fit the following mass-balance equation of glucosamine hexamer hydrolysis:

$$\begin{array}{l} 6\;\times \;{\left[{\left(\mathrm{GlcN}\right)}_{6}\right]}_{\mathrm{initial}}=2\;\times \;\left[{\left(\mathrm{GlcN}\right)}_{2}\right]+3\;\times \;\left[{\left(\mathrm{GlcN}\right)}_{3}\right]\\ \;\;+4\;\times \;\left[{\left(\mathrm{GlcN}\right)}_{4}\right]+6\;\times \;\left[{\left(\mathrm{GlcN}\right)}_{6}\right] \end{array}$$

From these corrected concentrations, the frequencies proportions of symmetrical and asymmetrical cleavage of (GlcN)_6_, as well as the tetramer re-cleavage, at the stages of about 20 up to 80% of substrate consumption, were calculated as follows :

frequency of cleavage of tetramer: (“2+2” cleavage) = ([dimer]-[tetramer])/3

frequency of symmetrical cleavage: (“3+3” cleavage + “2+2” cleavage) = [trimer]/2 + ([dimer]-[tetramer])/3

frequency of asymmetrical cleavage: (“4+2” cleavage) = [tetramer] + frequency of “2+2” cleavage

### M**ass spectrometric setup**

Directly after mixing substrate and enzyme, each mixture was filled into a syringe. The ‘reactor’ syringe (Hamilton-Bonaduz, Switzerland, 500 μL) located in a syringe pump (model 11 Plus, Harvard Apparatus, Hugo Sachs Elektronik, Hugstetten, Germany) infused the reaction mixture continuously at a flow rate of 5 μL/min (Tubing: 1/16”× ID 0.13 mm, length 200 mm) via an electrospray ionization source into the mass spectrometer like described previously [25,27]. Individual measurements were carried out at 20°C ± 2°C. The detection was performed in positive ionization mode with a Time-of-Flight (ToF) mass spectrometer from Agilent (Santa Clara, USA), model 6210 Time-of-Flight LC/MS. The most important MS parameters are 300°C drying gas temperature, 480 Lh^-1^ drying gas flow rate, 15 psig nebulizer gas pressure, 4000 V capillary voltage, 60 V skimmer voltage and 150 V fragmentor voltage, respectively. The mass-range was set to 160 – 3200 m/z and data acquisition was 0.88 cycles/sec. For the system control and data acquisition an Agilent Technologies (Waldbronn, Germany) software was used (Analyst QS, LC-MS TOF Software, Ver. A.01.00 (B663), Edition: June, 2004).

### Thermal unfolding experiments

#### Thermal unfolding experiments in presence or absence of (GlcN)_3_

To obtain the thermal unfolding curve of the enzyme protein, the CD value at 222 nm was monitored, while the solution temperature was raised at a rate of 1°C/min by a temperature controller (PTC-423L, Jasco). The buffer used was 50 mM sodium acetate buffer, pH 5.5. The final concentrations were 2.3 μM for the enzyme and 2.3 mM for (GlcN)_3_. To facilitate comparison between unfolding curves, the experimental data were normalized as follows. The fraction of unfolded protein at each temperature was calculated from the CD value by linearly extrapolating the pre- and post-transition baselines into the transition zone, and plotted against the temperature. Assays were performed in duplicate. Thermodynamic parameters could not be obtained, because of the poor reversibility of the unfolding transition.

#### Thermal unfolding experiments in presence or absence of chitosan

Intrinsic tryptophan fluorescence was used to measure the folding state of chitosanases. Thermal unfolding curves were obtained as previously described [28]. Sufficient quantities of chitosanase (3.0-8.5 μg) were added to 4-ml quartz cuvette to obtain 900–1000 relative fluorescence unit (RFU). The buffer used was 50 mM sodium acetate buffer, pH 5.5. Chitosan was used at the final concentration of 500 μg/ml.

## Results

### Choice of Arg42 for site-specific saturation mutagenesis

**Figure 1:**
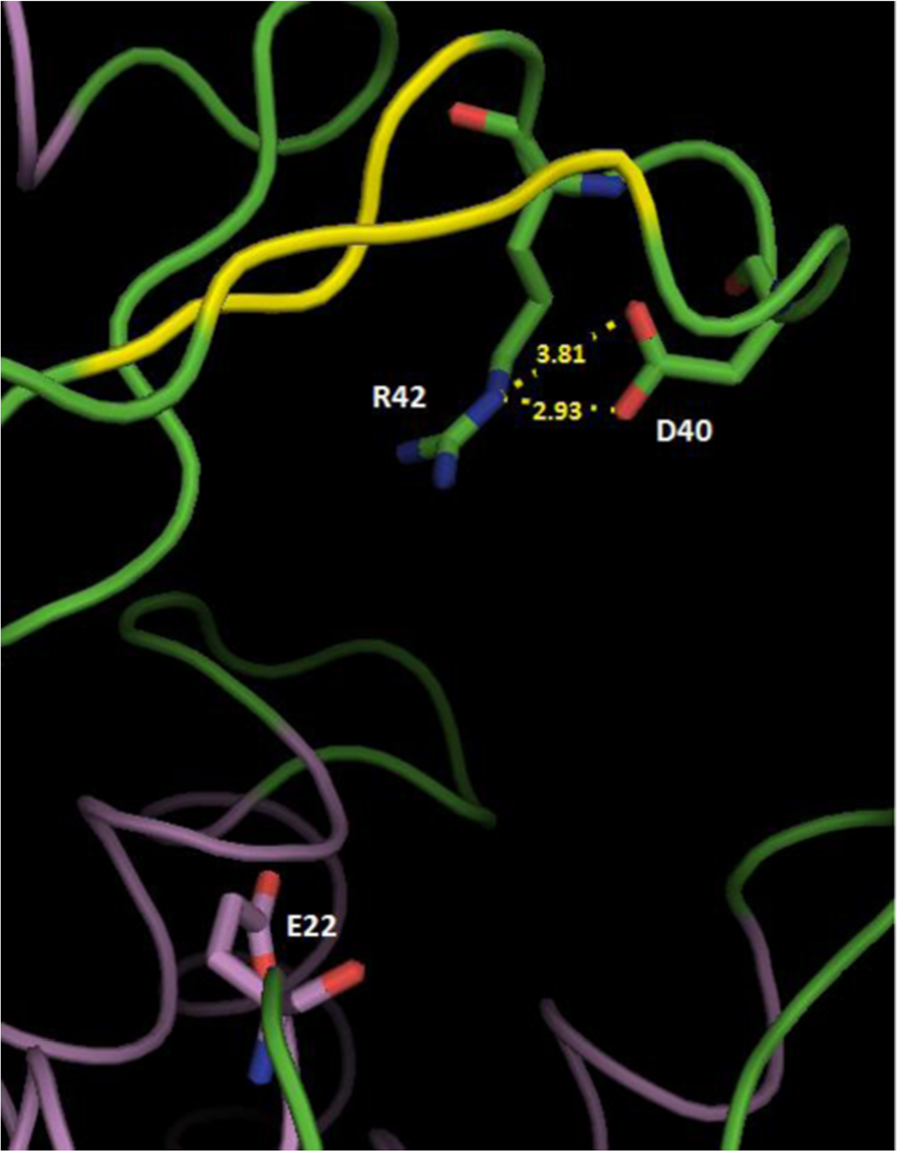
**Structural view of a part of the active site cleft of chitosanase CsnN174.** The image represents a portion of the chain A from 1CHK file in Protein Data Bank [6]. Catalytic residues: Glu22 (general acid), Asp40 (general base). Relevant interatomic distances in Å are indicated by interrupted yellow lines. The model was drawn using PyMOL software (PyMOL version 0.99, DeLano Scientific LLC, South San Francisco, CA, USA).

To find out if there were any residues that could possibly assist Asp40 to achieve its function as catalytic general base, we first examined the 3D structure of the catalytic cleft of *Streptomyces* sp. N174 chitosanase (CsnN174) [6]. It resulted that Asp40 is located very close to the side chain of Arg42: the nϵ atom of the guanidyl substituent in Arg42 is 2.93 Å from the Oδ2 atom of the Asp40 carboxylate group. Also, the side chain of Arg42 points towards the catalytic cleft of the chitosanase (Figure 1).

This close proximity suggests a possible interaction between Asp40 and Arg42. The structure-based alignment of the primary sequence of CsnN174 with the other members of GH46 family revealed that the arginine residue at position 42 is conserved in all the chitosanases biochemically characterized [17] strongly suggesting that this arginine plays an important role in catalysis [3]. This arginine is listed in the molecular signature of glycoside hydrolase families 46 and 80 [10] in the PROSITE database [29].

### Chitosanase assays of Arg42-mutated chitosanases from proteins extracts from *E. coli* cells

**Figure 2:**
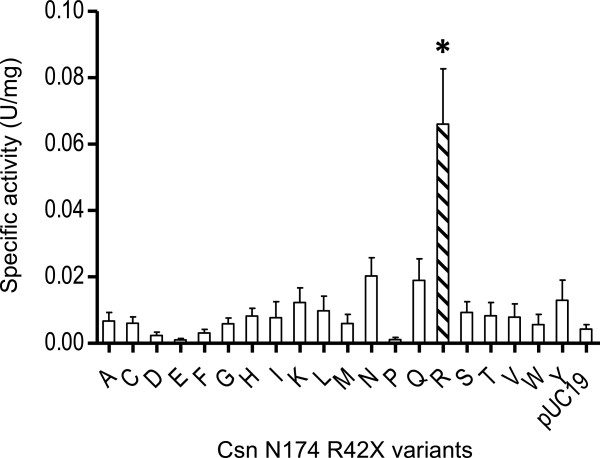
**Specific activity of CsnN174 R42X mutants in crude extract of *E. coli *cells.** (*) indicates that the specific activity significantly differs from that directed by the empty pUC19 vector (ANOVA test, *P*<0.05).

To assess the impact of Arg42 mutations on the catalytic activity of CsnN174, this residue was substituted by all the 19 other amino acids found in proteins. All mutated genes were expressed in *E. coli* cells using induction with 1 mM IPTG. Chitosanase activities were assayed directly from whole cell crude protein extracts. These preliminary results showed that any mutation in Arg42 resulted in a considerable loss of enzymatic activity (Figure 2).

### Specific activities and kinetic analysis of purified Arg42-mutated chitosanases toward chitosan

To better understand the implication of Arg42 in the catalytic mechanism of chitosanase, we first purified two mutated chitosanases, R42E and R42K, in which the strongly basic Arg42 residue was replaced by acidic (Glu) or less basic (Lys) residue, respectively. Specific activities obtained for these enzymes are listed in Table 1.

**Table 1:** Specific activities of purified wild-type and mutant CsnN174

**Enzyme**	**Specific activity (units per mg protein)**
Wild type*	52.9
D40G*	1.6
R42E	0.9
R42K	1.4
D40G+R42E	0.0012
D40G+R42K	0.018

* Data from ref. [17].

Substitutions of Arg42 by Glu or Lys severely affected the catalytic activity. As shown in Table 1, R42E and R42K mutants retained, respectively, 1.7% and 2.6% of wild type specific activity. The drastic loss of activity when Arg42 is mutated not only to an acidic residue (Glu), but also into a basic one (Lys), strongly suggests that Arg42 might be essential to maintain CsnN174 catalytic activity.

In our previous work, we have shown that the D40G mutant retains significant enzymatic activity (3% of wild type specific activity) despite the lack of the general base residue [17]. Because of the possible interaction between Asp40 and Arg42, we also purified two double mutated chitosanases in which the D40G mutation was accompanied by a mutation of Arg42 into Glu (D40G+R42E) or Lys (D40G+R42K).

The two double mutants D40G+R42E and D40G+R42K retained, respectively, only 0.075% and 1% of the D40G mutant activity (0.002% and 0.03% of wild type activity) (Table 1). These data show that Arg42 is essential for catalytic activity even in the chitosanase whose active center has been reconfigured by the D40G mutation. The severe loss of catalytic activity in both the wild type and D40G configuration of CsnN174 suggests that the role of Arg42 might arise not only from its interaction with Asp40. Thus, Arg42 must also accomplish another role in CsnN174 than that of interacting with Asp40. Because of the severe loss of activity caused by Arg42 substitution in the D40G context, D40G+R42E and D40G+R42K mutants were not further investigated.

We determined optimum pH for chitosanase activity of R42E and R42K mutants. However, optimum pH values of both mutants did not differ from the optimum pH 5.5 value for wild-type chitosanase (data not shown).

**Figure 3:**
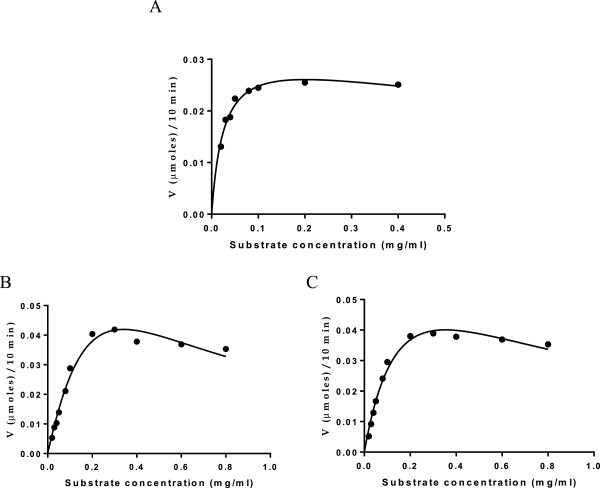
**Effect of substrate concentration on wild type or mutant chitosanase activity.(A)** wild type. **(B)** R42E mutant. **(C)** R42K mutant. Experimental data were interpreted according to a substrate inhibition model.

Because substrate inhibition was previously observed for CsnN174 and other chitosanases not only for high molecular weight chitosan [1,30] but also for glucosamine oligosaccharides [31], kinetic parameters were obtained for wild-type and mutant chitosanases and interpreted according to the substrate inhibition model (Table 2 and Figure 3).

**Table 2:** Kinetic parameters of purified wild-type and mutant CsnN174

**Enzyme**	***K***_**m **_**(μg/ml)**	***k***_**cat **_**(min**^**-1**^**)**	***K***_**i **_**(μg/ml)**	***k***_**cat**_**/ *K***_**m **_(min*ml/μg)
Wild-type	26.1	642.1	1547	24.6
R42E	441.7	96.7	263	0.22
R42K	204.1	55.5	612	0.27

Analysis of kinetic parameters obtained for the Arg42 mutants revealed that the turnover rates of R42E and R42K, as reflected in their *k*_cat_ values, were, respectively, 1/7 and 1/12 of the wild type chitosanase. Both mutants also had higher *K*_m_ values than the wild type chitosanase (17-fold increase for R42E and 8-fold increase for R42K compared to wild type). Thus, the substitution of Arg42 had an important effect on the substrate-binding mode of chitosanase. The combination of the decrease in *k*_cat_ and the increase in *K*_m_, resulted in decreases of overall catalytic efficiency (*k*_cat_/*K*_m_) of R42E and R42K respectively of 111-fold and 91-fold as compared to the wild type, which is consistent with the specific activities observed for these mutants. As mentioned above, kinetics of the hydrolysis of chitosan of CsnN174 wild type and both Arg42 mutants did not exhibit the classical Michaelis-Menten fitting curve, but rather followed a model assuming that substrate inhibition influenced the rate of chitosanase–catalyzed reaction. Non-linear regression curve fitting yielded a *K*_*i*_ value of 1547 μg/ml for *wt* CsnN174, a substrate concentration 59-fold greater than the *K*_m_ value. In the case of R42E and R42K mutants, the *K*_*i*_ values were, respectively, of 263 μg/ml and 612 μg/ml which are 6-fold lower for R42E and 2.5-fold lower for R42K than that of wild type. These data showed that substrate inhibition was relatively low for wild type CsnN174, being only observed at substrate concentrations well above the *K*_m_ value, whereas Arg42-mutated chitosanases were rapidly inhibited as substrate concentration was increased. Furthermore, the *K*_*i*_ value was even below the *K*_m_ value (441.7 μg/ml) when Arg42 was substituted by the strongly acidic residue, glutamate. Both mutated chitosanases were inhibited by increasing substrate concentrations to a greater extent than the wild type chitosanase.

Overall, kinetic analysis showed that R42 mutants were impaired in their catalytic activity as well as in their interaction with substrate.

### Thermal unfolding of the Arg42-mutated chitosanases

**Figure 4:**
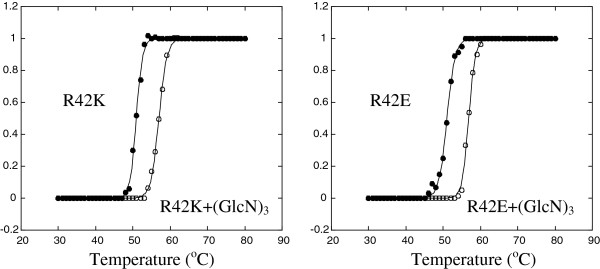
**Thermal unfolding curves of mutant chitosanases in the absence or presence of (GlcN)**_**3**_**. (A)** R42K chitosanase. **(B)** R42E chitosanase. The unfolding process was monitored by CD at 222 nm.

**Figure 5:**
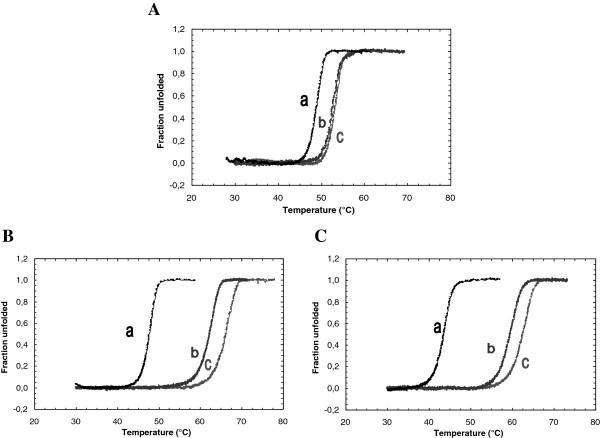
**Thermal unfolding curves of wild type or mutant chitosanases in the absence or presence of chitosan. (A)** wild type. **(B)** R42K chitosanase. **(C)** R42E chitosanase. **(a)** no chitosan. **(b)** 82.5% DDA chitosan. **(c)** 99% DDA chitosan. The unfolding process was monitored by intrinsic tryptophan fluorescence.

As reflected by the changes in *K*_m_ values, the substitution of Arg42 by glutamate or lysine seemed to affect the enzyme affinity to the substrate. Hence, the oligosaccharide binding ability of the Arg42 mutant chitosanases was evaluated by thermal unfolding experiments. The thermal stability of chitosanases in absence or presence of (GlcN)_3_ was assessed by monitoring CD at 222 nm (Figure 4). In the presence of (GlcN)_3_, the transition temperatures (*T*_*m*_) values of R42E and R42K mutant chitosanases increased by 5.8°C and 6.1°C, respectively (Figure 5). These *T*_*m*_ elevations upon the addition of (GlcN)_3_ were highly comparable to that of 5.7°C observed for the wild type enzyme [17]. Therefore, (GlcN)_3_ binding enhanced the protein stability of both Arg-42 mutants in a similar extent to that observed for the wild type chitosanase.

**Figure 6:**
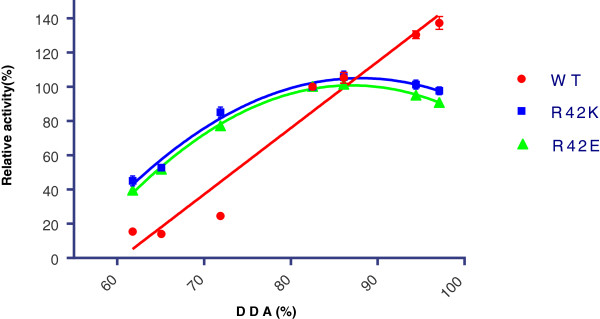
**Effect of the degree of *N*-deacetylation of chitosan on wild-type and Arg42-mutated chitosanases activity.** Wild-type or mutated chitosanases have been incubated with various chitosans in standard conditions and activity was measured by reducing sugar assay. For each enzyme, the activity against Sigma chitosan (84%-*N*-deacetylated) was taken as a reference (100%).

As previously reported by Roy *et al.* [2007], fluorometric determination of *T*_*m*_ in presence of chitosan can provide additional information about the mechanism by which mutations can influence chitosanase thermal stability. Thus, chitosan binding ability of the Arg42 mutant chitosanases was also assessed by monitoring intrinsic tryptophan fluorescence (Figure 6). An essential condition for using enzyme-substrate fluorometric experiments is that assays must be done under non-limiting condition with respect to substrate concentration [28]. As a consequence, chitosan concentration of 500 μg/ml was used in order to avoid substrate depletion under the experimental conditions. The *T*_*m*_ values of chitosanases in the presence or absence of two chitosan samples differing by their degrees of deacetylation (DDA) were determined as listed in Table 3. In the absence of chitosan, *T*_*m*_ of the R42K mutant was only slightly lower than that of wild type chitosanase. The replacement of Arg42 by a lysine did not affect significantly the thermal stability of this mutant. The substitution of Arg42 by a glutamate resulted in a greater decrease of *T*_*m*_ (Table 3). Possibly, the replacement of Arg42 by a glutamate disrupted some inter-residue interactions resulting in a decrease of thermal stability. More significantly, the *T*_*m*_ elevations due to contact with 84%-deacetylated chitosan were, respectively, almost 4-fold and 3-fold higher for mutated enzymes than that observed for wild type chitosanase. The *T*_*m*_ elevations upon the addition of chitosan were even higher for the mutated enzymes when tested with 99%-deacetylated chitosan (Figure 5 and Table 3), while the wild type chitosanase did not show further *T*_*m*_ elevation when put in the presence of this highly deacetylated substrate. Globally, chitosan binding elevated the *T*_*m*_s of both mutants to a much greater extent than that observed for the wild type chitosanase, suggesting that the mutation of the Arg42 residue significantly affected the binding mode of chitosan substrate to chitosanase.

**Table 3:** **Transition temperatures of thermal unfolding of wild type and R42-mutated CsnN174 chitosanases in the absence or presence of two chitosans with different degrees of*N*-deacetylation**

		***T***_***m***_**(°C)**			
**Enzyme**	**Enzyme only**	**Enzyme + chitosan (DDA 84%)**	**Enzyme + chitosan (DDA 99%)**	**Chitosan (DDA 84%)**	**Chitosan (DDA 99%)**
WT	48.8	53.4	53.2	4.6	4.4
R42E	43.2	59.5	62.5	16.3	19.3
R42K	47.7	62.5	66.1	14.8	18.4

### Effect of the degree of *N*-deacetylation of chitosan on the activity of Arg42-mutated chitosanases

We also investigated the activity of the various chitosanases with respect to the degree of *N*-deacetylation of their substrates (in the 62% - 97% DDA range; Table 4). The relative initial activities (with activity against Sigma chitosan taken as 100%) are shown on Figure 6. For wild-type chitosanase, a clear preference was observed for substrates with high degrees of *N*-deacetylation. However, a different pattern was observed for Arg42-mutated chitosanases: the chitosan with 86% DDA was the best substrate for R42K and R42E mutants (Table 4 and Figure 6) while activities slightly decreased against highly *N*-deacetylated chitosans. Moreover, the Arg42 mutant enzymes remained relatively more active than wild-type chitosanase against less *N*-deacetylated chitosans (Figure 6). As a result, wild-type chitosanase was 9-fold more active on 97% *N*-deacetylated chitosan than on 62% *N*-deacetylated chitosan while R42K and R42E mutants were only 2-fold more active on the latter (Table 4). Thus, mutations of R42 residue had a pronounced effect on substrate preference of chitosanase.

**Table 4:** **Specific activities of wild type and mutated CsnN174 chitosanases towards chitosans with different degrees of*N*-deacetylation**

	**Specific activity of chitosanase (U/mg)**		
**Chitosan degree of*N*-deacetylation (%)**	**WT**	**R42K**	**R42E**
97	73± 2	1.37 ± 0.03	0.816 ± 0.006
94	69 ± 1	1.42 ± 0.04	0.854 ± 0.008
86	56 ± 2	1.48 ± 0.05	0.91 ± 0.01
84	52.9	1.4	0.9
72	13.0 ± 0.6	1.19 ± 0.04	0.69 ± 0.01
65	7.5 ± 0.9	0.73 ± 0.01	0.464 ± 0.005
62	8.1 ± 0.5	0.63 ± 0.04	0.35 ± 0.01

### Analysis of (GlcN)_6_ hydrolysis by the Arg42-mutated chitosanases

**Figure 7:**
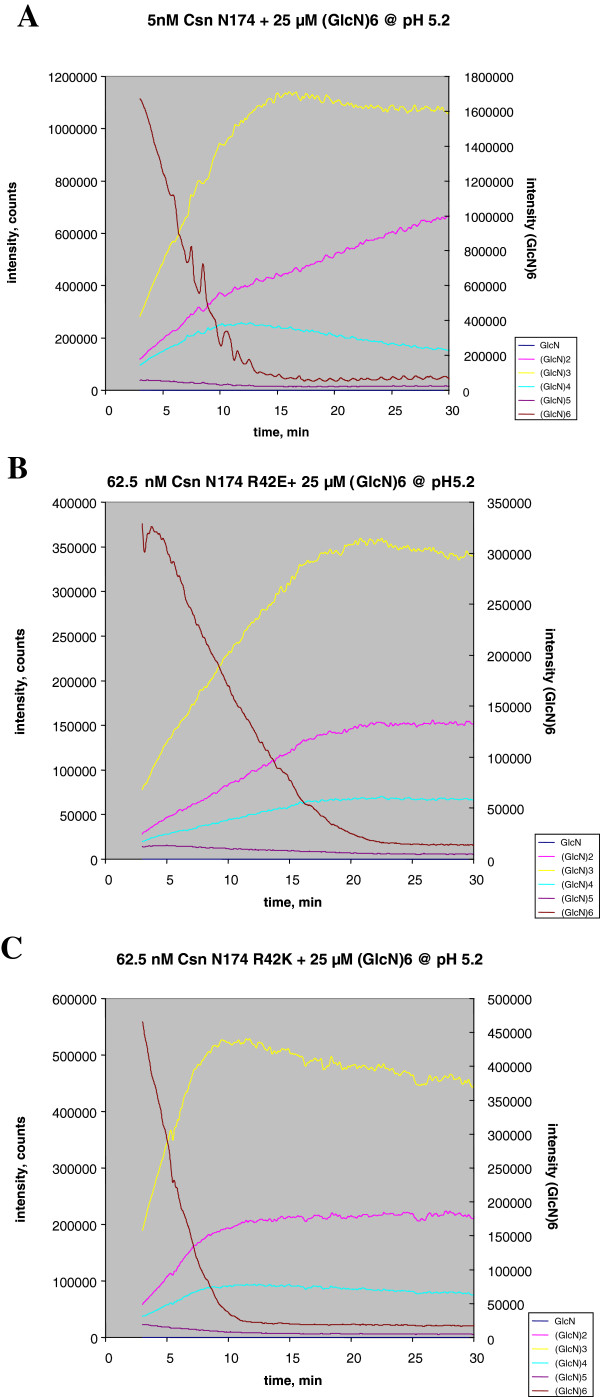
**Time-courses of (GlcN)**_**6 **_**hydrolysis catalyzed by wild-type, R42K and R42E chitosanases monitored by real-time mass spectrometry.** The enzymatic reactions were carried out in 10 mM ammonium acetate-containing aqueous solutions pH 5.2 at 20°C. **A)** (GlcN)_6_ hydrolysis time-courses obtained for wild type endochitosanase (5.0 nM)-catalyzed reaction performed with 25.0 μM of the substrate (GlcN)_6_; **B)** (GlcN)_6_ hydrolysis time-courses obtained for R42K chitosanase (62.5 nM)-catalyzed reaction performed with 25.0 μM of the substrate (GlcN)_6_; **C)** (GlcN)_6_ hydrolysis time-courses obtained for R42E chitosanase (5.0 nM)-catalyzed reaction performed with 25.0 μM of the substrate (GlcN)_6._

To further understand the interaction of the Arg42 mutant enzymes with the substrate, the mode of hydrolysis of (GlcN)_6_ was also investigated. The reaction time-courses of (GlcN)_6_ hydrolysis by R42E and R42K mutant enzymes were monitored by real-time MS [27] and the profiles of product production were analyzed. The specific activities of the mutant chitosanases were determined from the degradation rate of (GlcN)_6_ (Figure 7) and were found to be 17 min^-1^ for R42E and 36 min^-1^ for R42K, which corresponded to ~5% and ~10% of the wild-type specific activity (333 min^-1^) – values which are consistent with those measured with high molecular weight chitosan.

As shown in Figure 7, the overall product distributions of the Arg-42 mutants were not changed when compared to that of wild-type [(GlcN)_3_ >> (GlcN)_2_ > (GlcN)_4_]. Nevertheless, closer examination of the product distribution during the reaction showed that the cleavage preferences of R42E and R42K mutant chitosanases differ from that of wild type (Figure 7 and Table 5).

**Table 5:** Relative cleavage frequencies of wild type and mutated CsnN174 chitosanases from (GlcN)_6_ hydrolysis at 50% of substrate depletion calculated from data on Figure 7

	**Symmetrical cleavage**			**Asymmetrical cleavage**
**Enzyme**	**6 → 3 + 3**	**4 → 2 + 2**	**Total**	**6 →4 + 2**
WT	5,14 (60%)	0,28 (3%)	5,42 (63%)	3,19 (37%)
R42E	5,66 (66%)	0,65 (8%)	6,21 (74%)	2,23 (26%)
R42K	5,73 (65%)	0,53 (6%)	6,26 (71%)	2,60 (29%)

Calculations are shown for the reaction stage at 50% substrate depletion (Table 5), but the observed trends were present during the reaction time-course from 20% up to 80% of substrate depletion as well (data not shown). First, the frequencies of symmetrical cleavage of hexamers into trimers were enhanced for the two Arg42 mutants in comparison to the wild-type enzyme. The increase of trimeric product formation in mutant-catalyzed hydrolysis was counterbalanced by a reduction of the asymmetric cleavage of (GlcN)_6_ into (GlcN)_4_ + (GlcN)_2._ Furthermore, the efficiency of the Arg42 mutant chitosanases to use (GlcN)_4_ as a substrate was greatly improved compared to that of the wild-type. When the cleavage frequency of (GlcN)_4_ into 2(GlcN)_2_ was calculated at the stage of 50% substrate hydrolysis, the cleavage frequency was of 0.65 for R42E and 0.53 for R42K (representing a 2.3-fold and 1.9-fold increase, respectively, compared to wild type). Overall, both Arg42 mutants seemed to favor symmetrical cleavage of hexasaccharide or tetrasaccharide at the expense of asymmetrical cleavage. Mutations of Arg42 altered the pattern of hydrolysis, suggesting again some role in interaction with the substrate.

## Discussion

Site-directed mutagenesis [32] is now used universally to identify essential amino acids for enzymatic catalysis. This technique revealed however an extremely complex picture of the enzyme mechanisms. Indeed, today we know that some residues other than the catalytic amino acids play an essential role in maintaining the integrity of the enzymatic function. The case of glycoside hydrolases does not make an exception: in the past few years, several studies showed that, apart from the carboxylic catalytic residues, other residues are crucial for catalysis [33-35]. In *Streptomyces* sp. N174 chitosanase, some key residues have been identified and are believed to modulate the p*K*_a_ of catalytic residues [9], to orient the nucleophilic water molecule in an appropriate way to attack the glycosidic bond [17], to stabilize the protein structure [36] and to participate in the substrate-enzyme interactions [11,21]. In this study, we demonstrated that the arginine 42 is important for the enzymatic function of CsnN174. Moreover, we showed that this arginine plays a dual role by influencing not only the catalytic nucleophile residue Asp40 but also by modulating the substrate binding mode.

The substitution of Arg42 by any other amino acid resulted in an important decrease of specific activity (Figure 2 and Table 1). The effect of replacement of this arginine was quite obvious in R42E and R42K as reflected by their severe loss of specific activity. This loss of activity can be partly explained by the drastic decreases of the *k*_cat_ values observed for these mutants (Table 2). Arg42 might be involved in creating the suitable electrostatic environment required for Asp40 to achieve its catalytic function. Examination of the protein structure with the what if program [37,38] showed that Arg42 and Asp40 are close enough to possibly interact through electrostatic type of interaction (Figure 1). In the case of the *Bacillus circulans* xylanase (GH11 family), it has been shown that the presence of a positive charge of an arginine in the vicinity of the catalytic nucleophile glutamate resulted in lowering the p*K*_a_ of the latter, consequently helping it to maintain a negatively charged state [39]. Substitution of the arginine by a lysine in this xylanase had only a slight effect on enzyme activity, while its substitution by a non-charged side chain residue (Asn) resulted in a dramatic loss of activity. In our case, both substitutions by lysine or glutamate resulted in severe loss of activity.

The role of the equivalent residue Arg57 in the chitosanase from *Bacillus circulans* MH-K1 has been investigated [15]. Mutations of this residue into alanine or glutamine resulted in abolition of the enzymatic activity. The authors concluded that this residue deprotonates the catalytic nucleophile Asp55 (equivalent of Asp40 in N174 chitosanase), a suggestion compatible with the participation of this arginine in an electrostatic interaction with the catalytic nucleophile.

However, our data suggest that Arg42 could have another function in the enzyme mechanism. The enhanced *K*_m_ values of Arg42 mutants indicate that they might be impaired in their substrate binding mode (Table 2). The fact that hydrolysis of chitosan by R42E and R42K chitosanases was markedly more affected by substrate inhibition than the wild type chitosanase, as reflected by the *K*_*i*_ values (Table 2), is also in agreement with this hypothesis. Analysis of the reaction time-courses of (GlcN)_6_ degradation by R42E and R42K mutants chitosanases showed that their cleavage patterns were significantly different from the wild-type enzyme. Both mutants favored symmetrical cleavage (GlcN)_3_ + (GlcN)_3_ at the expense of the asymmetrical one generating (GlcN)_4_ + (GlcN)_2._ Furthermore, the relative frequency of (GlcN)_4_ cleavage into (GlcN)_2_ + (GlcN)_2_ was enhanced compared with wild-type (Table 5). Implication of Arg42 in substrate binding was also reflected by *T*_*m*_ elevations upon the addition of chitosan (Table 3), much higher in mutants than for wild type enzyme; an effect further amplified by the high degree of *N*-deacetylation. However, implication of Arg42 in substrate binding was not reflected by *T*_*m*_ elevations upon the addition of (GlcN)_3_, an oligosaccharide which is not further hydrolyzed by the enzyme (Figure 4). Because the substrate binding cleft of chitosanase is described by a symmetrical model including six subsites (−3)(−2)(−1)(+1)(+2)(+3) with cleavage occurring in the middle [4], trisaccharide binding must occur completely either on a side, or other of the cleavage site [2,21]. Therefore Arg42 residue seems to influence the productive binding of substrate but not the non-productive binding as exemplified by interaction with (GlcN)_3_.

These observations correlate well with the change in profile of activity against chitosans with various degrees of *N*-deacetylation (Table 5) resulting from mutation of Arg42. The effect of the degree of *N*-deacetylation of chitosan on chitosanase initial specific activity has been reported by several teams. The GH46 chitosanase from *Bukholderia gladioli* CHB101 hydrolysed chitosan of 70% degree of deacetylation to a higher extent than completely deacetylated chitosan [40]. Another GH46 family member, the chitosanase from *Streptomyces coelicolor* A3(2), was found to be more active against fully deacetylated chitosan [41]. Several chitosanases from GH8 family such as chitosanase from *Bacillus cereus* D-11 [42] and from *Bacillus* sp. P16 J [43] were most active against chitosans in the 80% to 90% *N*-deacetylation range. On the other hand, the GH8 chitosanase from *Paenibacillus* sp. 1794 was able to hydrolyse chitosans within a wide range of degrees of *N*-deacetylation (62% - 98%) with similar efficiency [30]. So far, it was then assumed in the literature that each enzyme has its own pattern of preference against the degree of *N*-deacetylation. We showed, for the first time, that this pattern can be modified by mutating a single residue. Considering the importance that the negatively charged carboxylic residues have in chitosan recognition by the chitosanases, we suggest than one important function of Arg42 is to optimize the electrostatic interaction between the charged residues of the substrate binding cleft and the amino-groups of GlcN residues in chitosan (positively charged at acidic pH). Mutation of Arg42 disrupted this charge equilibrium, and, as a consequence, an altered interaction between enzyme and substrate was observed, resulting in decreased activity (especially for highly *N*-deacetylated substrates), enhanced substrate inhibition and different positioning of oligosaccharide substrates. The substrate inhibition may also suggest the access of more than one molecule of substrate, which leads to binding of a second substrate molecule to the active site, forming a nonproductive substrate-enzyme-substrate complex [44,45].

## Conclusions

In this study, we demonstrated that the residue Arg42, highly conserved in GH46 family, substantially contributes to the enzymatic function of the *Streptomyces* sp. N174 chitosanase. The assignment of a specific function to Arg42 could arise from the determination of the structure of the enzyme-substrate complex. However, the predictions based on the structure of the free enzyme are of limited value for GH46 chitosanases, as the available experimental data suggest that the enzyme belonging to this family must undergo substantial conformational change while binding the substrate, narrowing the catalytic cleft in order to achieve catalysis [2,11,17,46]. In the context of an enzyme-substrate complex structure, interaction between Arg42 and Thr45 (a residue though to orient the nucleophilic water molecule involved in the catalytic mechanism [17]) could also be expected. Nuclear magnetic resonance spectroscopy experiments are progress and will hopefully give rise to new information on the enzyme-substrate complex structure [2].

## Abbreviations

CD: Circular dichroism; Csn N174: Chitosanase from *Streptomyces* sp. N174; ESI-MS: Electrospray ionization-mass spectrometry; GH: Glycoside hydrolase family; GlcN: D-glucosamine; (GlcN)n: β-D-glucosamine oligosaccharide with n monomer units; IPTG: Isopropyl-β-D-1-thiogalactopyranoside.

## Competing interests

The authors declare that they have no competing interests.

## Authors’ contributions

RB and MELH designed the study. MELH carried out all genetic biomolecular manipulations, enzyme purification and kinetic studies. MF participated to enzyme purification and performed the thermal unfolding experiments in presence of chitosan. TO, TF and TL carried out the biophysical experiments and their analysis. All authors co-wrote the manuscript and approved its final form.
